# Supplementary material for: TRPA1 Polymorphisms Modify the Hypotensive Responses to Propofol with No Change in Nitrite or Nitrate Levels
Source: Curr Issues Mol Biol. 2022 Dec 14;44(12):6333–45. doi: 10.3390/cimb44120432 (PMC9777046; doi:10.3390/cimb44120432)
Supplement: Supplementary file 1 [file cimb-44-00432-s001.zip › Supplementary Table S1.pdf]

**Supplementary Table S1.** Genotype and Haplotypes frequencies for *TRPA1* polymorphisms

| Polymorphisms/haplotypes | **N** | **%** |
|---|---|---|
| **rs920829** | | |
| CC | 127 | 77. 4 |
| TC | 35 | 21.3 |
| TT | ----- | ------ |
| Allele T | 289 | 89.1 |
| Allele C | 35 | 10.8 |
| **rs16937976** | | |
| CC | 119 | 72.5 |
| CG | 37 | 22.5 |
| GG | 7 | 4.2 |
| Allele C | 275 | 84.3 |
| Allele G | 51 | 15.6 |
| **rs13218757** | | |
| GG | 120 | 73.1 |
| AG | 35 | 21.3 |
| AA | 7 | 4.2 |
| Allele G | 275 | 84.8 |
| Allele A | 49 | 15.1 |
| **Haplótipos** | | |
| CCG | 243 | 74.5 |
| CGA | 46 | 14.1 |
| TCG | 32 | 9.8 |
