# Supplementary material for: TRPA1 Polymorphisms Modify the Hypotensive Responses to Propofol with No Change in Nitrite or Nitrate Levels
Source: Curr Issues Mol Biol. 2022 Dec 14;44(12):6333–45. doi: 10.3390/cimb44120432 (PMC9777046; doi:10.3390/cimb44120432)
Supplement: Supplementary file 1 [file cimb-44-00432-s001.zip › Supplementary Table S2.pdf]

Supplementary Table S2– Effect of *TRPA1* genotypes on changes in heart rate induced by propofol after adjustment for selected variables.

	HR (bpm)		
		R^2=0.25	RMSE=11.10
**Source**	β	95% CI	P
Age (years)	-0.10	-0.20 to 0.00	0.034*
BMI (kg/m2)	-0.09	-0.40 to 0.20	0.525
Use of ACEi	-1.70	-5.66 to 2.23	0.394
BBP	-0.40	-0.49 to -0.31	<0.001*
**genotypes**[a]			
rs920829[a]	+3.31	-1.18 to 7.82	0.148
rs16937976[b]	+5.52	-17.29 to 28.34	0.633
rs13218757[c]	-4.01	-27.04 to 19.01	0.731

Abbreviations: **BMI**-Body Mass Index; **β-** Parameter estimate; **CI**- confidence interval; **HR**- heart rate; **BBP**- Basal blood pressure. [a]Reference genotype: CC ; [b]Reference genotype: CC; [c] Reference genotype: GG. * p<0.05.
