# Supplementary figures and images for: TRPA1 Polymorphisms Modify the Hypotensive Responses to Propofol with No Change in Nitrite or Nitrate Levels
Source: Curr Issues Mol Biol. 2022 Dec 14;44(12):6333–45. doi: 10.3390/cimb44120432 (PMC9777046; doi:10.3390/cimb44120432)

**A** **rs920829**

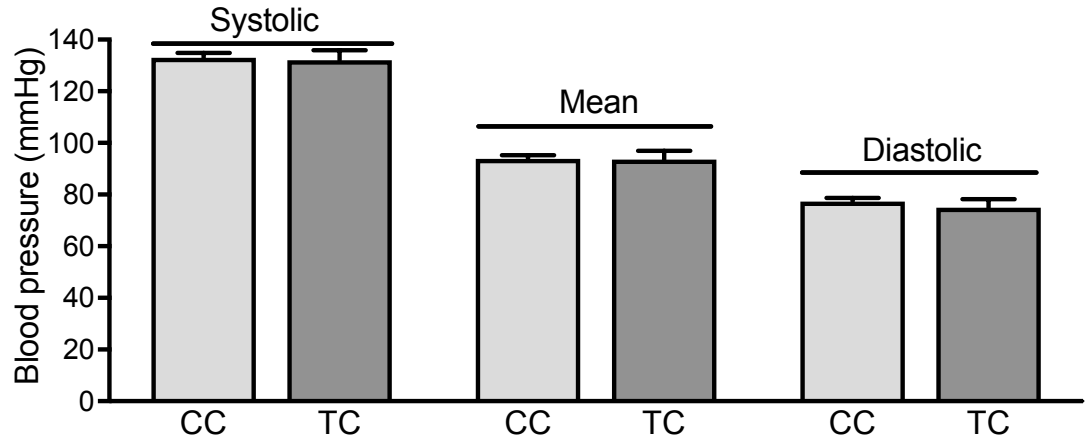

**B** **rs16937976**

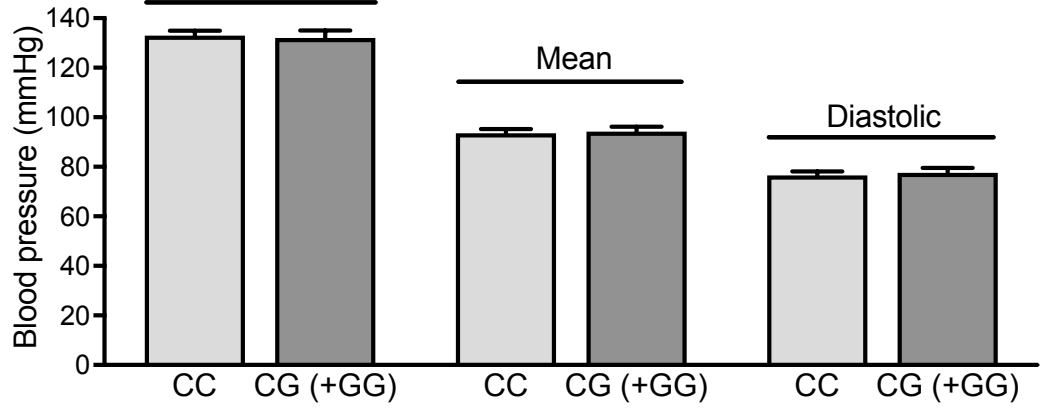

**C** **rs13218757**

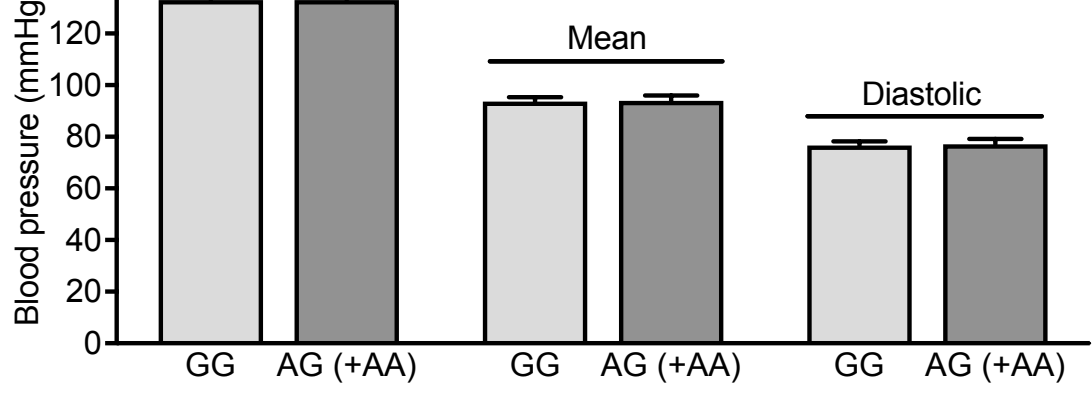

**D** **Haplotypes**

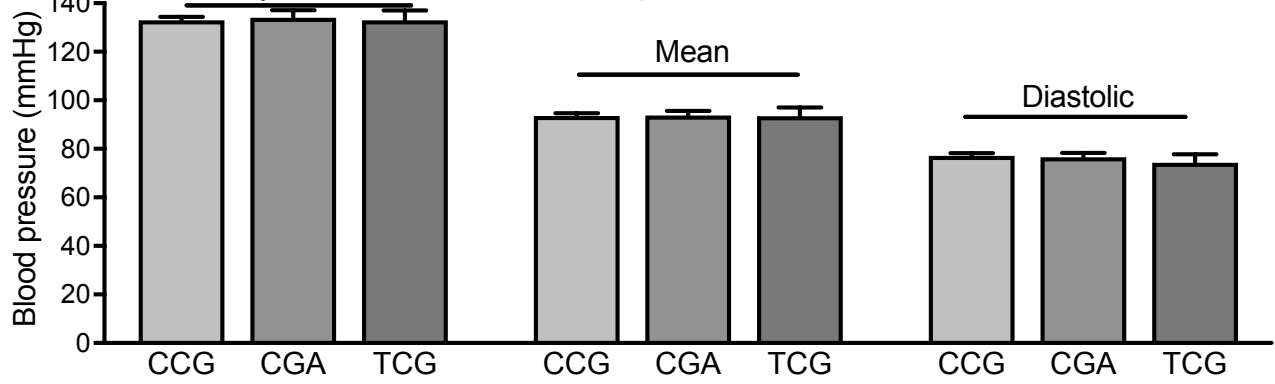

Supplement: Supplementary file 1 [file cimb-44-00432-s001.zip › Supplementary Figure S1.pdf]
